# Artificial intelligence (AI) adoption and Chinese art teachers’ career exploration behavior: The roles of job substitution risks, job insecurity, and organizational AI readiness

**DOI:** 10.1371/journal.pone.0362478

**Published:** 2026-11-06

**Authors:** Jia Li, Yun Gao

**Affiliations:** School of Culture, Tourism and Arts, Shanxi Vocational University of Engineering Science and Technology, Jinzhong, China; Prague University of Economics and Business: Vysoka Skola Ekonomicka v Praze, CZECHIA

## Abstract

The increasing use of artificial intelligence (AI) in art education raises questions about teachers’ professional security and career development. Drawing primarily on Conservation of Resources (COR) theory and, in a complementary manner, on Career Construction Theory (CCT), this cross-sectional study examined associations among AI adoption, perceived job substitution risk, job insecurity, career exploration behavior, and organizational AI readiness. Survey data from 381 Chinese art teachers were analyzed using Mplus 7.4. AI adoption was positively associated with career exploration behavior. Statistically significant indirect associations were observed through job substitution risk, job insecurity, and their hypothesized sequential combination. Organizational AI readiness positively moderated the AI adoption–career exploration relationship: the association was stronger at higher readiness. COR theory offers a resource-threat interpretation of the indirect associations, while CCT highlights that career exploration may reflect threat-related coping, growth-oriented development, or both. The data did not separately measure these motivations or establish causal ordering. The findings provide context-specific insights into career exploration among art teachers during AI-related technological change.

## 1. Introduction

The rapid integration of Artificial Intelligence (AI) into the workplace represents a profound technological shift. In the educational sector, the adoption of specialized AI tools is actively transforming instructional methods and curricular content [1], and this technological disruption introduces significant uncertainty, notably the perceived risk that AI could substitute for core teaching tasks, thereby threatening educators’ traditional roles and job security [2,3]. While creative professions requiring high emotional investment and original thinking, such as artistic creation, have traditionally been considered at lower risk of AI substitution [4,5], contemporary AI technologies, exemplified by AIGC, have demonstrated robust capabilities in image generation, style imitation, and even providing creative inspiration, which may challenge the core competencies underpinning art teachers’ professional authority and their established professional identity [5,6]. Unlike technologies primarily used to automate routine instructional or administrative tasks, generative AI can produce visual artworks that overlap with activities central to art teachers’ creative expertise. These capabilities may raise concerns about the distinctive value of teachers’ aesthetic judgment, artistic identity, and role in evaluating creative work [7,8]. Consequently, understanding how employees, specifically art teachers who face this dual pressure of technological transformation and identity redefinition, adapt professionally through behaviors such as career exploration is a critical and timely inquiry.

Current research on workplace AI has predominantly focused on its impact on general employee well-being and attitudes. Studies have established links between AI-related perceptions and outcomes such as increased anxiety, turnover intention, psychological distress, and workplace cynicism [9–11]. However, the existing literature has largely examined passive or negative reactions (e.g., distress, avoidance) and has paid insufficient attention to proactive career adaptation behaviors. Career exploration—a purposeful set of activities aimed at gathering information about career options and possibilities [12]—may represent an important adaptive response to technological change, yet the possible diversity of its underlying motivations remains insufficiently considered in research on AI and teaching [13].

However, career exploration activities may reflect different purposes even when the observable behaviors are similar. Some teachers may investigate alternative career paths in response to concerns about employment security, the declining value of established skills, or threats to professional identity (threat-driven exploration). Others may explore emerging career possibilities out of curiosity or aspirations for professional growth (growth-driven exploration). These motivations may coexist. For art teachers, generative AI may raise concerns about traditional artistic expertise while also creating opportunities in AI-assisted creative practice, digital pedagogy, and interdisciplinary roles. Distinguishing these possibilities helps avoid treating all career exploration as a uniform response to technological threats.

Despite the growing literature on AI-related occupational uncertainty, less is known about how AI adoption is associated with career exploration among art teachers and how organizational AI readiness may condition this relationship. To address these gaps, this study asks: How is AI adoption associated with Chinese art teachers’ career exploration behavior? By focusing on this question, the study examines the professional implications of AI in art education and considers ways to support educators navigating technological transitions.

## 2. Theoretical review and research hypotheses

### 2.1. Theoretical framework

This study is based on Conservation of Resources (COR) theory, which proposes that individuals strive to acquire, retain, and protect valued resources [14–16]. Potential losses of job security, professional standing, or occupational identity can be associated with stress and with efforts to protect or replenish resources [17]. Guided by COR theory, the proposed model examines the associations of AI adoption with career exploration behavior among Chinese art teachers. AI adoption is operationalized as teachers’ individual use of AI tools, rather than direct exposure to an externally imposed stressor. Nevertheless, this use occurs amid a broader technological transformation that may make the potential automation of artistic and teaching tasks more salient. Within the proposed model, job substitution risk represents an appraisal of possible task replacement and skill devaluation [18,19], whereas job insecurity represents concerns about future employment stability [20,21]. These perceptions may be associated with career exploration, including information seeking about alternative occupational opportunities [22]. Organizational AI readiness, reflected in institutional infrastructure, training, and supportive practices, constitutes a contextual resource that may condition the AI adoption–career exploration association [23–25].

Career Construction Theory (CCT) complements this resource-oriented account by explaining how people adapt to career transitions while constructing and reconstructing their vocational self-concepts [26]. The career construction model distinguishes adaptability resources from adapting responses; exploration of possible career directions is one such response [27,28]. For art teachers encountering generative AI, exploration may be interpreted as threat-driven when it concerns protecting career security and professional identity, or growth-driven when it concerns discovering new creative, pedagogical, or interdisciplinary opportunities. These motivations can coexist and are not necessarily identifiable from the same outward exploratory actions. COR remains the principal rationale for the model’s associations involving perceived job substitution risk and job insecurity, whereas CCT informs the interpretation of the meaning of career exploration. The study measures overall exploration behavior and does not separately test these proposed motivational orientations.

The proposed framework therefore specifies theoretically ordered associations among AI adoption, perceived job substitution risk, job insecurity, and career exploration behavior. This ordering is consistent with a possible resource-threat appraisal and adaptive-response account, but the cross-sectional data cannot determine its temporal or causal direction. Organizational AI readiness is examined as a boundary condition that may shape the strength of the association between AI adoption and career exploration. Adding CCT clarifies why career exploration cannot be assumed to be exclusively an anxiety-driven response: similar behaviors may also be relevant to ongoing professional development and vocational self-construction.

### 2.2. Application of AI and career exploration behavior

Based on COR theory, when major AI-related technological changes occur in the workplace, individuals may perceive threats to their valued resources (such as job security and professional identity) and engage in adaptive actions to manage potential resource losses [14–16]. The widespread use of AI technologies in educational settings—including generative AI tools, intelligent tutoring systems, and automated assessment platforms—is fundamentally changing teaching methods, skill needs, and professional relationships for Chinese art teachers [29,30]. For art teachers, this technological shift not only alters traditional teaching methods but may also be perceived as a threat to their expertise and job security, raising concerns about AI-related job replacement [31]. According to COR theory, such perceived threats may generate psychological pressure and uncertainty about future career opportunities [17], which may be associated with adaptive career behaviors.

Within the COR framework, perceptions of environmental threats and resource loss act as key precursors to career adaptation behaviors [14]. In the context of AI integration in education, when art teachers worry that AI tools may diminish their professional value or replace their teaching duties, they may become more inclined to actively seek information about educational trends, develop new skills, explore interdisciplinary opportunities, and consider alternative career options [13]. Such exploration behaviors, whether cognitive (e.g., re-evaluating career directions) or practical (e.g., pursuing professional development, researching alternative career paths), may serve as strategic coping mechanisms to reduce anxiety and regain control over their professional lives [32,33]. From a CCT perspective, career exploration may reflect both efforts to manage occupational threats and aspirations for professional growth [26,27]. For art teachers, such exploration may include investigating opportunities in AI-assisted artistic creation, digital arts education, and interdisciplinary professional development. Therefore, in the specific context of art education, AI adoption may be positively associated with career exploration as teachers respond to perceived challenges and emerging career opportunities.

Consequently, this study proposes:

H1: AI adoption is positively related to Chinese art teachers’ career exploration behavior.

### 2.3. The mediating role of job substitution risk

COR theory offers a fundamental framework for understanding how individuals aim to protect valued resources, with perceived threats to these resources potentially generating stress responses [16]. Technological disruptions, such as AI adoption, may heighten feelings of vulnerability among employees, particularly when AI technologies demonstrate the ability to perform tasks traditionally undertaken by humans [34]. In the specific context of AI adoption in education, technologies such as generative AI art tools and intelligent tutoring systems may be perceived as threats to key resources for Chinese art teachers, including job security, professional identity, and the relevance of their artistic skills [18]. This technological change fundamentally reshapes teaching workflows and redefines professional boundaries, making AI integration a relevant context for educators to evaluate potential resource losses [29,30]. A key mechanism in this process is the perception of job substitution risk. When art teachers believe that AI applications could automate their core instructional tasks or lessen the value of their artistic expertise, they may engage in cognitive assessments of potential resource loss. Such appraisals reflect concerns that their professional roles could become increasingly replaceable as AI capabilities develop [35]. These perceptions may signal potential skill devaluation and threats to valued occupational resources [19]. Within art education, this is especially relevant. AI tools capable of creating artwork or offering automated feedback may be perceived as challenges to the creative contributions and professional roles of art teachers [4]. In particular, AI-generated imagery and artistic style imitation overlap with creative activities central to teachers’ artistic expertise, potentially raising concerns about the continuing value of their aesthetic judgment and evaluative roles [6,8]. Therefore, this study proposes:

H2a: AI adoption is positively related to job substitution risk.

As mentioned above, job substitution risk reflects the belief that teaching tasks could be automated or devalued by AI [35,36]. These perceptions may threaten the perceived value of existing professional skills and resources, encouraging efforts to identify alternative career possibilities [31,37]. According to COR theory, individuals facing threats to valued resources may engage in behaviors aimed at acquiring new resources and restoring balance [14]. Career exploration—conceptualized as purposeful actions and cognitions aimed at acquiring information about careers, jobs, and organizations [12]—may serve as an adaptive mechanism for navigating professional uncertainty during technological disruption [32]. Within this framework, job substitution risk may be associated with career exploration behaviors, such as seeking AI-related training, exploring alternative career paths, or developing digital skills [4]. For Chinese art teachers, whose traditional instructional roles may be challenged by AI-generated artwork and automated teaching tools [3], perceptions of job substitution risk may be related to greater efforts to protect their professional futures. From a CCT perspective, such exploration may also involve reconsidering vocational identity and possible career directions in response to changing professional roles [26]. Related research on career adaptation and technological change suggests that perceived occupational threats may be associated with career-related responses [38,39]. For example, educators who perceive AI as a potential substitute for human instruction may investigate skill development opportunities or alternative professional directions [33,40]. Therefore, based on COR theory, job substitution risk is expected to be positively associated with career exploration behavior, consistent with efforts to manage potential losses of occupational resources. Accordingly, this study proposes:

H2b: Job substitution risk is positively associated with art teachers’ career exploration behavior.

Based on H2a and H2b, the study proposes H2: Job substitution risk mediates the relationship between AI adoption and Chinese art teachers’ career exploration behavior.

### 2.4. The mediating role of job insecurity

Job insecurity is defined as “a subjective anticipation of a fundamental and involuntary event related to job loss” [41] or, more broadly, as the experience of uncertainty about the future of one’s work [42]. It reflects a negative personal assessment of employment stability. According to COR theory, when people perceive that valued resources (such as stable employment and professional status) are threatened or decreasing, they may experience psychological stress associated with job insecurity [16]. In the educational setting, the adoption of AI—including generative AI art tools and intelligent tutoring systems—occurs within a technological environment that may challenge key teacher resources, such as job security, professional identity, and the value of specialized skills [18]. The widespread use of AI in art education changes teaching methods, questions the relevance of traditional artistic skills, and blurs professional boundaries, potentially increasing perceived occupational uncertainty among Chinese art teachers [31]. Specifically, AI adoption may make teachers more aware of the possibility that their teaching duties could be automated or devalued by AI [35]. When art teachers see AI’s ability to produce artistic content, offer automated feedback, or imitate creative styles [37], they may perceive potential threats to their distinctive creative roles and the value of their professional skills. In art education, concerns about the changing value of creative expertise, aesthetic judgment, and artistic evaluation may extend beyond individual tasks to broader uncertainty about professional roles and future employment stability. Such concerns are consistent with COR theory’s emphasis on threats to valued resources [43]. Studies also suggest that technological disruption may be associated with perceptions of occupational vulnerability and insecurity [44]. For example, teachers who perceive that AI could automate elements of their professional work may question the future stability of their employment. Therefore, within the COR framework, AI adoption is expected to be positively associated with job insecurity in the context of perceived occupational resource threats. Accordingly, this study proposes:

H3a: AI adoption is positively related to job insecurity.

Job insecurity is often associated with withdrawal and self-protective work behaviors. From the perspective of COR theory, job insecurity represents a perceived threat to important resources, such as job stability and safety, which may encourage individuals to conserve or acquire resources [16]. Extensive research links job insecurity to lower organizational commitment and trust [45,46], higher turnover intentions [47,48], and other career-related responses. Job insecurity may be accompanied by feelings of self-doubt, weakened professional identity, and uncertainty about future career opportunities [49]. According to COR theory, perceived threats to occupational resources may be associated with efforts to identify alternative opportunities. In this sense, job insecurity may encourage individuals to consider ways of managing employment-related uncertainty [50]. Career exploration is an active, problem-solving coping strategy aimed at regaining a sense of control by identifying new career options [51,52]. Specifically, career exploration involves actively seeking and assessing information about one’s interests, abilities, external job prospects, and organizational data through both thinking and practical actions [12]. Such activities may help individuals identify alternative career paths, consider relevant skills, and prepare for possible career transitions [32]. From a CCT perspective, career exploration can also be understood as an adapting response to occupational uncertainty, without assuming that all exploratory behavior is motivated exclusively by insecurity [26,27]. For Chinese art teachers, exploration may include investigating AI-assisted artistic creation, developing digital art competencies, considering opportunities in curriculum design or educational technology, or exploring other professional roles. Through such activities, teachers may identify potential career resources and reconsider their future professional directions [52]. Related research suggests that job insecurity may be associated with career-related responses and adaptation behaviors [47,50]. Therefore, in the context of AI adoption in art education, job insecurity is expected to be positively associated with career exploration as teachers consider ways to manage uncertainty about their professional futures. Accordingly, this study proposes:

H3b: Job insecurity is positively associated with art teachers’ career exploration behavior.

Based on H3a and H3b, this study proposes H3: Job insecurity mediates the relationship between AI adoption and Chinese art teachers’ career exploration behavior.

### 2.5. Job substitution risk and job insecurity

In the context of AI adoption in Chinese art education, specialized AI tools like generative art software and intelligent tutoring systems are actively transforming teaching practices and curriculum content [1]. As AI capabilities in visual creation, style imitation, and even critical analysis advance [53], teachers continually evaluate how their human-centric skills compare to AI’s growing abilities. Those who perceive a significant overlap and believe their core tasks are replaceable tend to feel greater anxiety and uncertainty about their professional future [54]. Additionally, greater awareness of AI’s expanding role in education may be associated with stronger perceptions of occupational threat and insecurity [38]. Such job insecurity may be accompanied by a reduced sense of control and predictability at work, as well as concerns about professional identity [41,55]. For Chinese art teachers, perceived job substitution risk is therefore expected to be positively associated with job insecurity. Accordingly, this study proposes H4b: Job substitution risk is positively associated with job insecurity among Chinese art teachers.

Based on H2a (H4a), H4b, and H3b (H4c), this study proposes H4: Job substitution risk and job insecurity serve as chain mediators in the relationship between AI adoption and Chinese art teachers’ career exploration behavior.

### 2.6. The moderating role of organizational AI readiness

Organizational AI readiness refers to an institution’s preparedness to adopt and integrate AI technologies through technological infrastructure, organizational capabilities, strategic alignment, and leadership support [56,57]. In educational settings, such readiness is reflected in access to AI-related resources, technical assistance, professional training, and institutional support for pedagogical innovation. These resources are particularly relevant to art teachers as AI increasingly reshapes artistic creation, instructional practices, and professional skill requirements. From the perspective of COR theory, individuals strive not only to protect existing resources but also to acquire and accumulate additional resources that facilitate adaptation to environmental change [16,58]. Accordingly, organizational AI readiness can be viewed as a contextual resource that facilitates technological adaptation while creating opportunities for professional learning and development. Consistent with this perspective, perceived organizational support may further strengthen employees’ perceptions that their contributions and professional development are valued [59].

Building on this resource-based perspective, organizational AI readiness may also shape how AI adoption translates into career exploration behavior. COR theory suggests that access to resources enables individuals to invest in activities that generate further resources, whereas resource scarcity may constrain such investments [14,58]. In institutions with higher AI readiness, art teachers who actively adopt AI technologies may therefore be better positioned to develop digital pedagogical competencies, engage in AI-assisted creative practices, and explore emerging professional opportunities. Career Construction Theory (CCT) complements this explanation by conceptualizing career adaptability as a set of psychosocial resources that enable individuals to respond to occupational change and construct future career possibilities [26,28]. Within this framework, career exploration represents an adapting response through which individuals investigate alternative opportunities and reconsider their professional trajectories. By facilitating resource acquisition and supporting career adaptation, higher organizational AI readiness may thus strengthen the positive association between AI adoption and career exploration behavior. Accordingly, the following hypothesis is proposed:

H5: Organizational AI readiness positively moderates the association between AI adoption and Chinese art teachers’ career exploration behavior.

The research model is shown in Fig 1.

**Fig 1:**
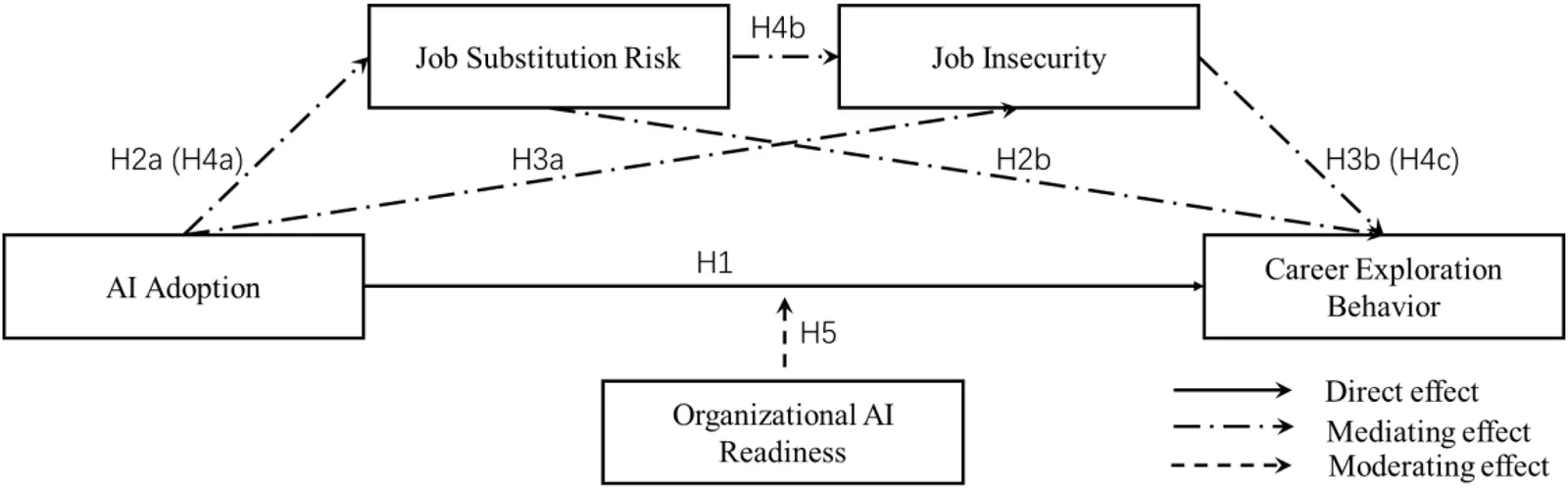
Research model is presented here for illustration.

## 3. Methods

### 3.1. Data collection

This study received ethical approval from the Human Research Ethics Committee of Shanxi Vocational University of Engineering Science and Technology (Approval Date: May 1, 2025; Approval Number: EA2025037). Data were collected using a structured questionnaire comprising an introduction outlining the study objectives, information on confidentiality and informed consent, and items assessing the study constructs. The measurement items were presented in random order to minimize potential response-order effects. To ensure linguistic and cultural appropriateness, items adapted from English-language instruments underwent a forward-translation and independent back-translation procedure [60,61]. Specifically, the original English items were first translated into Chinese, and the resulting Chinese versions were independently translated back into English. The back-translated items were compared with the original versions, and any discrepancies were reviewed and resolved to preserve semantic and conceptual equivalence while ensuring relevance to the Chinese art education context. Before finalization, the questionnaire was further refined through two rounds of feedback from art teachers to improve item clarity and contextual appropriateness.

From May 21 to June 4, 2025, a pre-test involving 60 teachers yielded 54 valid responses. Results showed satisfactory psychometric properties, with all constructs demonstrating Cronbach’s alpha values above 0.7, and all items exhibiting factor loadings greater than 0.6. Additionally, 90.7% of participants reported a clear understanding, and 94.4% completed the questionnaire within 5–7 minutes, confirming its reliability, validity, and suitability for formal research.

A formal questionnaire survey was conducted from July 17 to September 26 2025. To address geographical barriers, we used an online data collection method. After finalizing the list of universities, we used social networks to contact friends, classmates, and acquaintances who worked at those institutions. Before asking for their help, we discussed the purpose, process, and importance of the research in detail with them and provided all necessary information about the survey, including its content, expected completion time, and data protection measures. Once we received their consent, we shared the survey link and asked them to invite art teachers from their universities to participate. To boost response rates and motivate participation, we offered incentives: each respondent who completed the survey successfully received a small gift worth 10 RMB. We distributed 500 questionnaires and received 439 responses, resulting in a response rate of 87.8%. According to relevant Chinese laws, all workers are over 18 years old, so the responses in this study do not include minors. Additionally, all participants provided electronic informed consent.

For the 439 collected questionnaires, we referenced relevant literature [17,62] to screen out unreasonable responses based on the following criteria: first, all answer options were highly consistent; second, completion times were abnormal (less than 2 minutes or more than 10 minutes were considered abnormal); third, invalid questionnaires were caused by errors or missing information. Ultimately, 381 valid questionnaires were retained. The demographic data of these 381 teachers are shown in Table 1.

**Table 1:** Descriptive statistics of respondents.

Characteristic	Mean (SD)	Category	n	%
Gender	—	Male	161	42.3
		Female	220	57.7
Age (years)	34.5 (6.7)	≤30	86	22.6
		31–35	162	42.5
		36–40	98	25.7
		≥41	35	9.2
Educational attainment	—	Bachelor’s degree or below	257	67.5
		Master’s degree	124	32.5
Teaching experience (years)	8.4 (5.1)	0–5	129	33.9
		6–10	138	36.2
		≥11	114	29.9
School levels	—	Junior college / Vocational college	145	38.1
		General undergraduate university	146	38.3
		Key undergraduate university	90	23.6
Weekly frequency of AI use	—	Less than 3 times	95	24.9
		3–5 times	74	19.4
		6–10 times	61	16.0
		11–15 times	67	17.6
		16–20 times	46	12.1
		21 times or more	38	10.0

### 3.2. Measure

AI adoption was assessed using a 4-item scale adapted from Sheng et al. [63], measuring teachers’ self-reported individual use of AI tools in artistic and instructional activities.

Job substitution risk was assessed using a 5-item scale adapted from Parvez et al. [64] and Zhao and Wu [18], capturing teachers’ perceptions of the potential replacement of their professional tasks and roles by AI technologies.

Job insecurity was measured using a 3-item scale developed by Hellgren and Sverke [65], with item wording adapted to assess uncertainty about continued employment in art education in the context of AI-related technological change.

Organizational AI readiness was measured using a 4-item scale developed by Hossain et al. [24] and Yin et al. [66], capturing teachers’ perceptions of institutional financial resources, AI-related expertise, technological infrastructure, and leadership support for AI-assisted art teaching. This operationalization aligns with the study’s focus on individual-level perceptions and career exploration behavior.

Career exploration behavior was measured using a 6-item scale developed by Stumpf et al. [12]. Respondents were asked to indicate the extent to which they had engaged in career exploration activities during the preceding three months. The items covered the investigation of career possibilities, participation in career development activities, and information seeking about professional opportunities.

This study utilized a 7-point Likert scale to assess all items, with 1 representing strongly disagree and 7 indicating strongly agree. The complete wording of all 22 measurement items is presented in Table 2 to facilitate assessment of the construct’s operationalization and measurement content.

**Table 2:** Measurement items of the study constructs.

Construct	Code	Measurement item
AI Adoption (AIA)	AIA1	I frequently use AI tools (e.g., image generators, layout assistants) to obtain artistic instructional materials or teaching inspiration for my art classes.
	AIA2	I rely on AI assistance to complete certain stages of my art teaching projects (e.g., courseware design, student work evaluation references).
	AIA3	I engage in real-time interaction with AI technology to accomplish creative or instructional design tasks in my art teaching.
	AIA4	AI tools automatically execute some repetitive or procedural parts of my art teaching work (e.g., sorting and organizing teaching resources, generating basic lesson plan frameworks).
Job Substitution Risk (JSR)	JSR1	I believe that my role as an art teacher could be replaced by AI technology.
	JSR2	AI applications are likely to take over most of my daily art teaching tasks in the near future.
	JSR3	Compared with other occupations, art teachers face a higher risk of being substituted by AI.
	JSR4	The development of AI makes my current art teaching work more replaceable.
	JSR5	If AI is widely used in education, my position as an art teacher may no longer be needed.
Job Insecurity (JI)	JI1	I feel uneasy about the possibility of losing my art teaching job due to the growing adoption of AI in the education industry.
	JI2	There is a risk that I will have to leave my current art teaching job in the near future because of industry shifts driven by AI.
	JI3	I am worried that I might be pushed out of my art teaching job by AI-related changes before I am ready to leave.
Organizational AI Readiness (OAR)	OAR1	Our institution has sufficient financial resources to support AI-assisted art teaching and artistic projects.
	OAR2	Our institution has a skilled workforce with expertise in AI technology to support AI-assisted art teaching practices.
	OAR3	Our institution has a robust AI-based infrastructure, including all necessary IT components for AI-assisted art teaching.
	OAR4	The leadership of our institution provides strong support by allocating labor, financial, and material resources for AI-assisted art teaching initiatives.
Career Exploration Behavior (CEB)	CEB1	I have investigated other career possibilities within or related to the art education field (e.g., art training, cultural and creative product development).
	CEB2	I have attended career development programs or events relevant to art teachers.
	CEB3	I actively obtain information about the job market and opportunities for art education professionals.
	CEB4	I initiate conversations with experienced individuals to learn about career paths in art education and related fields.
	CEB5	I seek out information about specific art education-related jobs, institutions, or roles that interest me.
	CEB6	I actively look for information about different types of careers in the art education and creative fields (e.g., school art teacher, after-school art instructor, art curator).

### 3.3. Data analysis strategy

Data were analyzed using Mplus 7.4. Following assessment of the measurement model, mediation and moderation effects were examined simultaneously using an integrated latent-variable structural equation model with robust maximum-likelihood estimation and numerical integration. The model incorporated job substitution risk and job insecurity as parallel and sequential mediators, while organizational AI readiness moderated the direct association between AI adoption and career exploration behavior. The latent interaction was estimated using the XWITH procedure.

Gender, age, educational attainment, teaching experience, institutional level, and weekly frequency of AI use were included as control variables in all endogenous equations. Continuous controls were grand-mean centered, and categorical controls were dummy-coded. Indirect effects and conditional direct effects were estimated using unstandardized coefficients, with 95% confidence intervals calculated. Simple slopes were evaluated at the mean, and one standard deviation above and below the latent moderator mean, with scale transformations applied for graphical presentation. Given the cross-sectional design, indirect effects were interpreted as statistical associations rather than causal relationships.

### 3.4. Common method bias test

To assess potential common method variance, Harman’s single-factor test was conducted using all 22 measurement items. The largest eigenvalue of the item correlation matrix was 6.173, accounting for 28.06% of the total variance, below the commonly used 50% threshold. In addition, the single-factor model exhibited poor fit (*χ^2^*(209) = 2564.49, CFI = .404, TLI = .341, RMSEA = .172, SRMR = .150). These results suggest that no single factor dominated the survey responses.

## 4. Results

### 4.1. Reliability and validity

As shown in Table 3, all 22 standardized factor loadings were statistically significant (p < .001), ranging from  .72 to  .81. The corresponding squared multiple correlations (SMCs) ranged from  .52 to  .66. Composite reliability (CR) values ranged from  .82 to  .89, and average variance extracted (AVE) values ranged from  .57 to  .60, supporting satisfactory internal consistency and convergent validity. Furthermore, as presented in Table 4, the square roots of AVE for all five constructs ranged from  .76 to  .77, exceeding the absolute values of their corresponding interconstruct correlations, which ranged from −.19 to  .36. These results satisfy the Fornell–Larcker criterion and provide evidence of satisfactory discriminant validity among the study constructs.

**Table 3:** Results of CFA and reliability and validity testing.

Construct	Item	Estimate	S.E.	p	SMC	CR	AVE
AIA	AIA1	.80	.03	***	.63	.85	.58
	AIA2	.77	.03	***	.59		
	AIA3	.74	.03	***	.55		
	AIA4	.74	.03	***	.55		
JSR	JSR1	.76	.03	***	.58	.87	.57
	JSR2	.74	.03	***	.55		
	JSR3	.81	.02	***	.66		
	JSR4	.72	.03	***	.52		
	JSR5	.74	.03	***	.55		
JI	JI1	.78	.03	***	.61	.82	.60
	JI2	.78	.03	***	.60		
	JI3	.77	.03	***	.59		
OAR	OAR1	.79	.03	***	.63	.85	.58
	OAR2	.80	.02	***	.64		
	OAR3	.73	.03	***	.53		
	OAR4	.73	.03	***	.53		
CEB	CEB1	.80	.02	***	.63	.89	.58
	CEB2	.72	.03	***	.52		
	CEB3	.76	.02	***	.58		
	CEB4	.78	.02	***	.61		
	CEB5	.72	.03	***	.52		
	CEB6	.78	.02	***	.62		

Note 1: AIA = AI adoption; JSR = job substitution risk; JI = job insecurity; OAR = organizational AI readiness; CEB = career exploration behavior. The same below.

Note 2: ***= *p* <  .001, ** = *p* <  .01, * = *p* <  .05, the same below.

**Table 4:** Variables’ correlation coefficient and the square root of AVE.

Dim	Mean	SD	AVE	AIA	JSR	JI	OAR	CEB
AIA	4.80	1.09	.58	**.76**				
JSR	3.85	1.19	.57	.33***	**.76**			
JI	3.35	1.42	.60	.32***	.30***	**.77**		
OAR	5.27	.89	.58	.03	−.19***	−.13**	**.76**	
CEB	4.24	1.32	.58	.33***	.36***	.29***	.07	**.76**

The bold font represents the square root value of AVE, while the lower triangle displays the Pearson correlation coefficient.

### 4.2. Model fit

Table 5 shows the comparison results with different models. A detailed analysis indicates that all the fit indices of the five-factor model meet high standards. In fact, this model performs better than all other models considered. These results strongly indicate that the five-factor model is the best choice for adoption.

**Table 5:** Results of comparisons with alternative models.

Model	Factor	*χ* ^*2*^ */df*	*CFI*	*TLI*	*RMSEA*	*SRMR*
Single-Factor Model	AIA + JSR + JI + OAR + CEB	12.27	.40	.34	.17	.15
Two-Factor Model	(AIA + CEB), (JSR + JI + OAR)	9.03	.58	.53	.15	.14
Three-Factor Model	(AIA + OAR), (JSR + JI), CEB	6.81	.70	.66	.12	.12
Four-Factor Model	AIA, (JSR + JI), OAR, CEB	3.70	.86	.84	.08	.07
Five-Factor Model	AIA, JSR, JI, OAR, CEB	1.95	.95	.94	.05	.04

### 4.3. Mediating effect

As shown in Table 6, the structural path estimates revealed significant positive associations among the key variables. AI adoption was positively associated with job substitution risk (B = .40, SE = .07, p < .001) and job insecurity (B = .37, SE = .08, p < .001). Job substitution risk was also positively associated with job insecurity (B = .22, SE = .07, p = .002). Furthermore, both job substitution risk (B = .35, SE = .08, p < .001) and job insecurity (B = .22, SE = .06, p = .001) were positively associated with career exploration behavior.

**Table 6:** Test of mediating effect.

Effect	B	SE	p	95% CI	Hypothesis
Total effect^a^	.51	.07	< .001	[.37, .66]	
Direct effect^a^	.27	.08	.001	[.12, .43]	Support
Total indirect effect	.24	.04	< .001	[.15, .33]	
Indirect effect (H2)	.14	.04	< .001	[.07, .21]	Support
Indirect effect (H3)	.08	.03	.003	[.03, .14]	Support
Sequential indirect effect (H4)	.02	.01	.027	[.002, .036]	Support

Note. All estimates were obtained from the same integrated latent-variable structural equation model using robust maximum likelihood estimation. The 95% confidence intervals were calculated using the delta method. Total and direct effects were evaluated at the latent mean of OAR. All estimates were adjusted for the control variables specified in the model.

The total association between AI adoption and career exploration behavior was positive and significant (B = .51, SE = .07, 95% CI [.37,  .66]). The direct association remained significant after accounting for the mediating variables (B = .27, SE = .08, 95% CI [.12,  .43]). Both estimates were evaluated at the latent mean of organizational AI readiness, thereby supporting H1 at this level.

The total indirect association was significant (B = .24, SE = .04, 95% CI [.15,  .33]). Specifically, the indirect association through job substitution risk was significant (B = .14, SE = .04, 95% CI [.07,  .21]), supporting H2. Similarly, the indirect association through job insecurity was significant (B = .08, SE = .03, 95% CI [.03,  .14]), supporting H3. The sequential indirect association through job substitution risk and job insecurity was also significant (B = .02, SE = .01, 95% CI [.002,  .036]), supporting H4.

### 4.4. Moderating effect

Table 7 presents the moderation results obtained from the integrated latent-variable model. AI adoption was positively associated with career exploration behavior (B = .27, SE = .08, p < .001). Organizational AI readiness (B = .27, SE = .09, p = .004), job substitution risk (B = .35, SE = .08, p < .001), and job insecurity (B = .22, SE = .06, p < .001) were also positively associated with career exploration behavior. More importantly, the interaction between AI adoption and organizational AI readiness was positive and statistically significant (B = .36, SE = .09, p < .001), indicating that organizational AI readiness strengthened the positive association between AI adoption and career exploration behavior. Thus, H5 was supported.

**Table 7:** Test of moderating effect.

DV	IV	Estimate	S.E.	Est./S.E.	*p*	Hypothesis (Y/N)
CEB	AIA	.27	.08	3.46	< .001	—
	JSR	.35	.08	4.26	< .001	—
	JI	.22	.06	3.41	< .001	—
	OAR	.27	.09	2.85	.004	—
	AIA × OAR	.36	.09	3.98	<.001	H5 (Y)

Note. All reported coefficients are unstandardized latent-variable estimates, including the simple slopes described in the text. All estimates were adjusted for gender, age, educational attainment, teaching experience, institutional level, and weekly frequency of AI use. Categorical covariates were dummy-coded, with male, bachelor’s degree or below, junior/vocational college, and AI use fewer than three times per week serving as the respective reference categories. Age and teaching experience were grand-mean centered. For graphical presentation, the simple slopes in Fig 2 were transformed to the mean-item-score scale.

To further examine this interaction, simple slope analyses were conducted at low (−1 SD), mean, and high (+1 SD) levels of organizational AI readiness (OAR). These levels were defined using the latent standard deviation of OAR (SD = .808), rather than the observed composite-score standard deviation (SD = .89; Table 4). The results showed that the association between AI adoption and career exploration behavior was not statistically significant at low OAR (B = −.02, SE = .11, p = .853), whereas significant positive associations were observed at the mean (B = 0.27, SE = .08, p < .001) and high levels (B = .57, SE = .10, p < .001). These findings suggest that the positive association between AI adoption and career exploration behavior strengthened as organizational AI readiness increased (Fig 2). For graphical presentation, the latent-variable simple slopes were transformed to the mean-item-score scale using B_plot = B_latent × SD_AIA × λ̄_CEB, where B_latent denotes the unstandardized latent-variable simple slope, SD_AIA is the latent standard deviation of AI adoption (1.053), and λ̄_CEB is the mean factor loading of career exploration behavior (.922).

**Fig 2:**
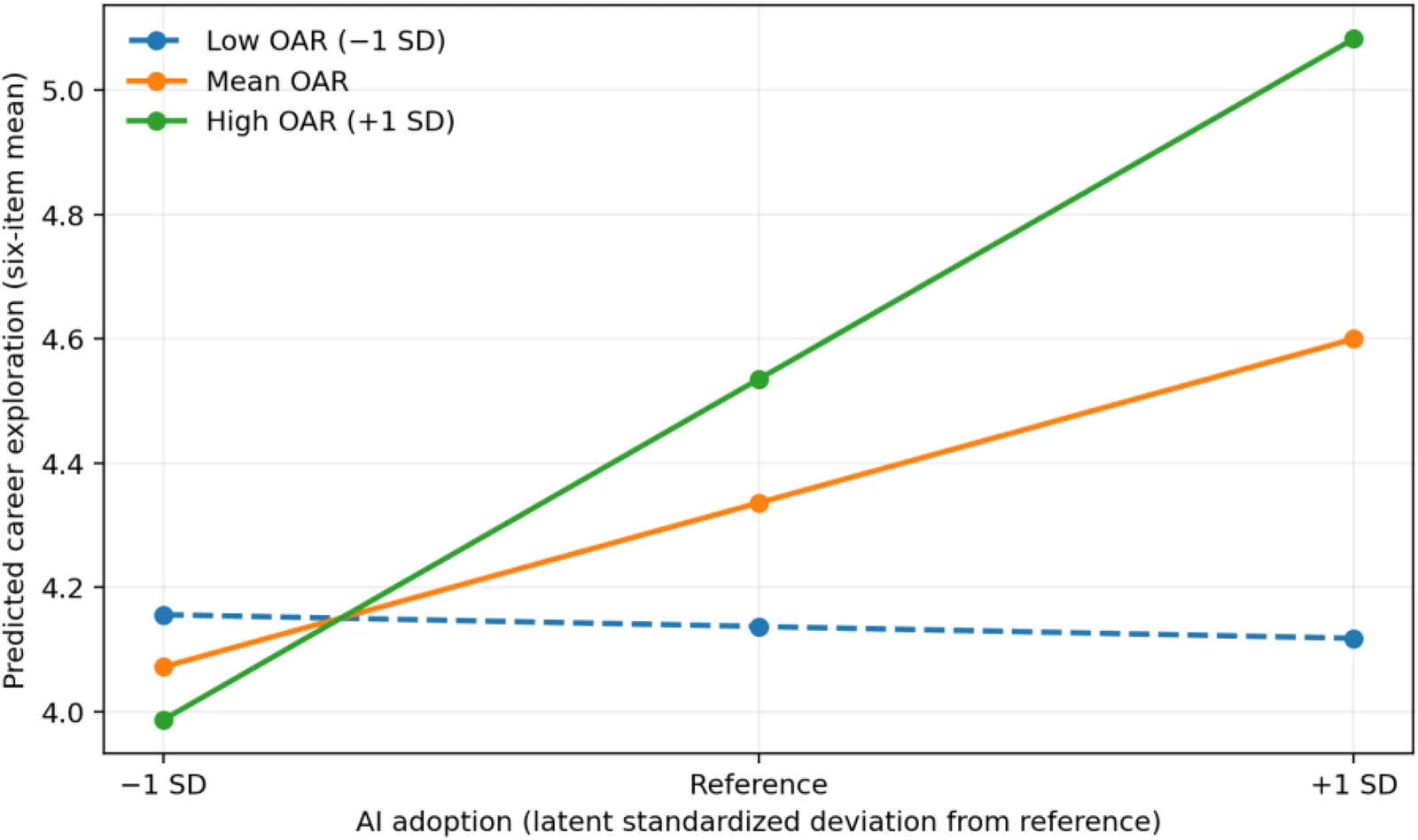
The moderating effect of OAR is presented here for illustration.

## 5. Discussion

First, AI adoption was positively associated with career exploration behavior. In art education, the use of generative AI tools may occur alongside concerns about teachers’ valued resources, including job security, pedagogical expertise, and creative authority [18,37]. Unlike technological applications primarily associated with routine task automation, generative AI may challenge the perceived distinctiveness of artistic expertise and aesthetic judgment because it can directly produce visual content and support creative processes [7,8]. COR theory suggests that perceived resource threats may be associated with efforts to seek other occupational possibilities or acquire career resources [12,14]. Nevertheless, individual AI use should not automatically be equated with an externally imposed threat: teachers may adopt AI for instructional benefits or professional curiosity as well as in response to uncertainty.

Second, the positive indirect associations involving job substitution risk and job insecurity are compatible with the proposed COR-based interpretation. Greater AI adoption was associated with greater perceptions of task-replacement risk, which were related to greater job insecurity and career exploration behavior. For art teachers, the ability of AI systems to generate images, imitate styles, or provide artistic feedback may make concerns about professional differentiation especially salient [19]. Such capabilities may raise concerns about the continuing value of teachers’ creative skills and their roles in interpreting and evaluating artistic work. In this context, job substitution risk reflects perceived threats to the distinctiveness of artistic and instructional tasks, whereas job insecurity concerns broader uncertainty about employment stability. A perceived threat to valued occupational resources provides a plausible account of these associations, but statistical indirect effects estimated from cross-sectional observations cannot demonstrate that substitution concerns arose before insecurity or exploration.

CCT provides an additional interpretive lens for the outcome of career exploration [26]. The positive indirect associations through job substitution risk and job insecurity are consistent with the possibility of threat-driven exploration, in which teachers consider career alternatives to manage uncertainty about their roles. Yet similar activities could be growth-driven: teachers may examine digital arts, AI-supported curriculum development, creative entrepreneurship, or interdisciplinary careers out of curiosity and aspirations for professional development. The present six-item career exploration measure records overall exploratory behavior and does not assess the reasons for exploring. Accordingly, neither the relative prevalence of these two motivations nor their distinct relationships with AI adoption can be established by these data. CCT broadens the interpretation of exploration; it does not supply evidence for two separately verified pathways [27,28].

Finally, organizational AI readiness positively moderated the association between AI adoption and career exploration behavior. The positive association was negligible at low readiness and stronger at high readiness. This pattern is consistent with H5, suggesting that organizational AI readiness may serve as a contextual resource associated with a stronger positive relationship between AI adoption and career exploration behavior. A plausible alternative is that supportive schools provide access to training, technical infrastructure, and developmental opportunities that facilitate career-related information seeking and experimentation [57,67]. For art teachers, these resources may also facilitate engagement with AI-assisted creative practices and emerging opportunities in digital arts education. From a CCT perspective, such exploration may be compatible with growth-oriented career adaptation. However, the study did not measure exploration motives or test whether institutional support operates through these proposed mechanisms. The result should therefore be interpreted as a positive statistical interaction rather than evidence of a specific motivational pathway.

### 5.1. Theoretical contribution

First, this study extends the application of COR theory to the intersection of AI adoption, art education, and career behavior [14,16]. Rather than treating teacher outcomes solely in terms of anxiety or intentions to leave, it examines career exploration as an adaptive response that may accompany perceptions of technological change [12,32]. The contribution lies primarily in examining career exploration as a meaningful career-development outcome, rather than claiming novelty for the general relationship between AI adoption and job insecurity. A complementary contribution is to incorporate CCT [26], which cautions against treating all exploration as homogeneous, defensive coping. Threat-driven exploration may express attempts to protect occupational resources, whereas growth-driven exploration may reflect curiosity, career development, and changes in vocational self-concept. This conceptual distinction is especially meaningful for art teachers because generative AI may challenge creative expertise and professional identity while also enabling novel artistic and pedagogical work [6]. These orientations were not separately operationalized or verified in the present study.

Second, the significant indirect associations through job substitution risk, job insecurity, and their hypothesized sequential combination provide evidence that these variables are statistically linked in the manner specified by the COR-based model. The findings are compatible with an account in which AI-related task-replacement concerns and employment uncertainty are associated with career exploration [19,35,41]. By situating these associations within art education, the study highlights occupational concerns involving creative expertise, aesthetic judgment, and professional identity, which may be particularly salient when AI can generate artistic content [6,8]. The findings do not demonstrate the causal sequence implied by the model arrows. CCT complements this explanation by identifying alternative motivations that may give otherwise similar exploration behaviors different meanings [27,28]. Consequently, the contribution lies in providing a more differentiated account of career-related associations during technological change rather than in establishing a uniform threat-induced behavioral mechanism.

Third, the positive interaction involving organizational AI readiness highlights the potential importance of institutional resources as a boundary condition [67]. Contrary to a simple threat-buffering expectation, higher organizational AI readiness was associated with a stronger positive link between AI adoption and career exploration behavior. One possible interpretation is that supportive environments not only alleviate some uncertainties but also create access to information, learning, and professional opportunities [57]. Related research among Chinese college teachers has also highlighted the relevance of workgroup emotional climate and individual work adaptation to innovative behavior [68]. For art teachers, such opportunities may involve AI-assisted artistic creation, digital pedagogy, and the development of new professional capabilities. This interpretation is consistent with a growth-oriented CCT perspective, but the present data do not establish the motivations responsible for the observed interaction. The finding thus extends the discussion of organizational resources beyond resource conservation while remaining appropriately cautious about unmeasured processes.

### 5.2. Practical implications

For educational institutions and school administrators, it is essential to go beyond simply deploying technology by establishing transparent communication channels and clearly emphasizing AI’s supportive role in teaching rather than viewing it as a threatening replacement. Such communication may help address teachers’ concerns about professional uncertainty. Additionally, administrators should focus on creating a supportive ecosystem. Specifically, schools should strengthen infrastructure that includes reliable hardware and technical support, design ongoing professional development programs tailored to arts education, and nurture an organizational culture that encourages innovation and experimentation. These forms of support may provide opportunities for teachers to pursue internal role development and teaching improvement, such as participating in AI-integrated curriculum design or interdisciplinary projects, while contributing to organizational learning and collective professional growth.

For education policymakers, we recommend developing practical guidance to support teachers during the integration of AI into art education. Such guidance could outline recommended institutional support measures, including psychological support, AI ethics training, and professional development resources. Furthermore, policymakers could consider incorporating AI literacy and application skills into art teacher preparation, professional qualification requirements, and relevant curriculum standards. These measures may help educational institutions create more supportive conditions for teachers’ professional adaptation and the integration of AI into art education.

For individual art teachers, engaging in structured professional learning and hands-on exploration—such as mastering AI-assisted creation tools, developing digital curation skills, or examining ethical issues related to AI in art education—may offer opportunities for professional growth during technological change. Such participation may also support the development of new competencies and encourage reflection on teachers’ evolving professional roles. Additionally, participating in peer learning communities can facilitate the exchange of experiences and resources related to AI integration, fostering collaborative learning and professional adaptation. Teachers can also communicate their resource and training needs to schools to help align institutional support with their professional development priorities.

### 5.3. Limitations and future directions

This study has several limitations worth noting. First, the use of cross-sectional data restricts causal inference. Although the model is grounded in COR theory, the relationships among AI adoption, perceived threats, and career exploration may be dynamic and reciprocal. For example, teachers who are already exploring alternative careers, particularly those who are proactive in professional development or possess stronger pre-existing digital competencies, may be more inclined to adopt AI tools. The estimated indirect associations cannot establish the assumed temporal ordering or rule out reverse causality and omitted common antecedents. Future research should use longitudinal designs with repeated measurements of AI adoption and career exploration to examine their potential bidirectional relationship, complemented by experimental or appropriately designed mixed-method approaches where feasible.

Second, the focus on Chinese art teachers provides context-specific insights but limits generalizability. Teachers’ experiences may depend on China’s institutional, policy, and cultural contexts. Recruitment through social contacts and referrals across selected universities may also have favored more connected or technologically engaged participants. Although aesthetic authority, creative identity, and evaluative expertise are relevant to art teaching, these concerns were not directly measured. Without a comparison group from other disciplines, the study cannot establish whether such concerns are more pronounced among art teachers. Future research should use more diverse recruitment strategies, include cross-disciplinary comparisons, and directly assess art-specific professional concerns across different educational and cultural settings.

Third, the six-item measure adapted from Stumpf et al. [12] assessed overall career exploration behavior rather than its underlying motivations. Although CCT provides a conceptual basis for distinguishing threat-driven and growth-driven exploration, the data cannot identify individual motives or determine how these orientations relate separately to job insecurity and organizational AI readiness. Future research should develop and validate motivation-specific measures and examine whether perceived threats, vocational curiosity, and growth opportunities have different antecedents and career consequences. Because the principal constructs relied on teacher self-reports, subsequent studies could also incorporate organizational records, independent assessments of AI readiness, and interviews to better distinguish perceived conditions from institutional practices.

Fourth, professional rank/title and pre-existing digital literacy/AI proficiency were not included as control variables. Professional rank may be associated with perceived job security, employment opportunities, and career mobility, while digital literacy and AI proficiency may shape technology adoption, perceived substitution risk, job insecurity, and career exploration. These characteristics may also be related to teachers’ age, professional experience, and organizational environment, making them relevant to the interpretation of the reported associations. Future research should measure professional rank and baseline digital competencies directly, include them as covariates where theoretically appropriate, and compare adjusted estimates with the original model to assess the robustness of the findings.

Fifth, the study relied on teacher-reported questionnaire data, which may be influenced by recall bias, social desirability, and common method variance. Although Harman’s single-factor test did not identify a dominant general factor, the possibility of shared-method effects remains. Moreover, organizational AI readiness reflects teachers’ perceptions rather than independently verified institutional conditions. In particular, the perceived sufficiency of financial resources was not assessed against standardized budgetary criteria. Similarly, self-reported career exploration may differ from objectively documented behavior. Future studies could combine teacher surveys with institutional financial records, AI-related training data, and independently verified indicators of career exploration to improve measurement accuracy.

## 6. Conclusions

This study examined associations among AI adoption, perceived job substitution risk, job insecurity, organizational AI readiness, and career exploration behavior in a cross-sectional sample of Chinese art teachers. Drawing on COR theory, the results showed that AI adoption was positively associated with career exploration and that significant indirect associations were present through job substitution risk, job insecurity, and their hypothesized serial combination. These findings are consistent with a possible resource-threat interpretation but do not determine causal or temporal ordering. Organizational AI readiness positively moderated the AI adoption–career exploration relationship, with a stronger positive association under higher readiness. The complementary CCT perspective highlights that career exploration may reflect efforts to manage occupational threats, to pursue growth opportunities, or both; this study did not separately measure these motives. Taken together, the findings suggest that educators’ responses to AI-related change should be understood in relation to perceived occupational risks and institutional developmental opportunities, while motivating further longitudinal and motivation-specific research.
