# Supplementary material for: Artificial intelligence (AI) adoption and Chinese art teachers’ career exploration behavior: The roles of job substitution risks, job insecurity, and organizational AI readiness
Source: PLoS One. 2026 Nov 6;21(11):e0362478. doi: 10.1371/journal.pone.0362478 (PMC13742945; doi:10.1371/journal.pone.0362478)
Supplement: Supplementary file 1 [file pone.0362478.s002.docx]

Dear editors and reviewers,

We would like to express our sincere gratitude for your invaluable guidance on our manuscript. Your comments are crucial to us and have significantly enhanced the quality of our work. Following your feedback, we have made further revisions to the initial manuscript, with ***all changes highlighted in yellow***. While the revised manuscript may still have some imperfections, we kindly ask for your ongoing suggestions to further improve it. Your continuous guidance is crucial for meeting the publication standards set by PLOS One. We once again thank you for your hard work and patient guidance. Below are the details of the amendments:

**Editor’s Comments**

Based on the reviews and my own reading of the manuscript, I am inviting you to submit a revised version that addresses the following major concerns. Please note that these revisions are necessary to ensure the manuscript meets **PLOS ONE’s publication criteria**, specifically Criterion 3 (high technical standard and sufficient detail) and Criterion 4 (conclusions are supported by the data).

**Comment 1:** **Methodological Rigor and Common Method Bias (Criterion 3)**
Because your study relies on cross-sectional, single-source, self-reported survey data, it is highly susceptible to Common Method Bias (CMB). Currently, the manuscript does not report any statistical tests to check for CMB. In your revision, please conduct and report appropriate tests (e.g., Harman’s single-factor test, or ideally, an unmeasured latent method construct in Mplus) to demonstrate that CMB does not invalidate your findings.

**Response 1:** Dear editor, thank you for your constructive comment regarding the potential influence of common method bias (CMB). We agree that the use of cross-sectional, single-source, self-reported survey data warrants careful consideration of possible method-related bias.

In response to your suggestion, we have added Section 3.4 (Common Method Bias Test) to report the results of Harman's single-factor test. The analysis included all 22 measurement items from the 381 respondents. The largest eigenvalue of the item correlation matrix was 6.173, accounting for 28.06% of the total variance, below the commonly used 50% threshold. In addition, the single-factor model exhibited poor fit (χ²(209) = 2564.49, CFI = .404, TLI = .341, RMSEA = .172, SRMR = .150).

These results suggest that no single factor dominated the survey responses, providing preliminary evidence that the observed relationships are unlikely to be explained solely by a single common factor.

We appreciate your valuable suggestion, which has helped us strengthen the methodological reporting and provide a more transparent assessment of potential bias in the study.

**Comment 2:** **Cross-Sectional Design and Causal Language (Criterion 4)**
Both the editorial review and Reviewer 2 noted that the cross-sectional nature of your data limits your ability to make causal claims, particularly regarding a chain mediation model. While you acknowledge this in the limitations, the manuscript frequently uses causal language (e.g., "triggers," "leads to," "develops into"). Please revise the manuscript throughout (especially in the Abstract and Discussion) to use associative language that accurately reflects the cross-sectional design. Furthermore, please address Reviewer 2’s valid concern regarding reverse causality: could proactive teachers who are already exploring alternative careers be more likely to adopt AI? This alternative explanation must be thoroughly discussed.

**Response 2:** Dear editor, thank you for your careful assessment and constructive guidance. We agree that the cross-sectional nature of our data does not permit causal conclusions, particularly regarding the temporal sequence implied by the proposed serial mediation model. We also appreciate your emphasis on potential reverse causality between AI adoption and career exploration behavior.

In response to your comment, we have reviewed the manuscript throughout and made targeted revisions to distinguish statistical associations from causal relationships.

First, we have refined the Abstract and Section 4.3 (Mediating Effect) to describe the findings in terms of observed associations and model-estimated indirect associations. In particular, the revised interpretation of the serial mediation results emphasizes that the statistically significant indirect pathways are consistent with the theoretically specified model but do not establish a causal sequence. We have also adjusted causal wording in Section 2.5 concerning the relationship between job substitution risk and job insecurity.

Second, we have revised Section 5.2 (Practical Implications) to ensure that the recommendations are presented as potential approaches for supporting teachers rather than as interventions whose effects have been established by the present study. The revised section retains the original recommendations for educational institutions, policymakers, and individual teachers while using more appropriately qualified language.

Third, we have carefully reviewed the Discussion and Theoretical Contribution sections, which already interpret the findings primarily in terms of observed associations and plausible theoretical explanations. For example, the Discussion explicitly acknowledges that the cross-sectional design does not establish a temporal process or causal sequence. We have therefore retained these interpretations to preserve the coherence of the manuscript.

Regarding reverse causality, Section 5.3 (Limitations and Future Directions) already explicitly acknowledges the alternative explanation raised by Reviewer 2. Specifically, we recognize that teachers who are already exploring alternative careers or developing new professional skills may be more inclined to adopt AI tools. The section also identifies pre-existing digital literacy and AI proficiency as unmeasured characteristics that may be associated with both technology adoption and career exploration. We further acknowledge that the estimated indirect associations cannot establish temporal ordering or exclude reverse causality and omitted common antecedents.

Accordingly, the manuscript recommends longitudinal, experimental, or appropriately designed mixed-method research to examine the direction of these relationships. The proposed ordering remains grounded in Conservation of Resources theory, while our empirical conclusions are limited to the associations supported by the cross-sectional data.

We sincerely appreciate your valuable guidance. These revisions have helped us improve the precision of our language and the interpretation of our findings while maintaining a coherent theoretical narrative. Your comments on alternative causal explanations have also provided valuable inspiration for the follow-up research we are currently planning.

**Comment 3: Construct Measurement and Translation (Criterion 3)**

- **Scale Translation:** You adapted scales from English literature (e.g., Stumpf et al., 1983) for a Chinese sample. Please explicitly describe the translation process (e.g., was a back-translation procedure used?) to ensure the validity of the instruments in the Chinese context.
- **Item Transparency:** As requested by Reviewer 2, please provide the full list of survey items used for each construct, either by integrating them into the main text (e.g., in a table) or clearly referencing them as a Supplementary File.
- **Construct Alignment:** The theory frames AI adoption as an "external stressor," but your sample item ("I frequently use AI tools...") measures voluntary, individual use. Please clarify this conceptualization. If AI adoption is a voluntary behavior, how does it function as an external threat?

**Response 3:** Dear editor, thank you for your careful review and constructive suggestions regarding scale translation, item transparency, and the conceptual alignment between AI adoption and the theoretical framework. We appreciate the opportunity to clarify these important methodological and conceptual issues.

First, regarding scale translation, we have expanded Section 3.1 (Data Collection) to provide a more detailed description of the translation and cultural adaptation procedures. Specifically, items adapted from English-language instruments were first translated into Chinese and then independently back-translated into English. The back-translated items were compared with their original versions, and discrepancies were reviewed and resolved to improve semantic and conceptual equivalence while ensuring appropriateness for the Chinese art education context. The questionnaire was further refined through two rounds of feedback from art teachers. We have also added relevant methodological references (Brislin, 1970; Beaton et al., 2000) to support the translation and cross-cultural adaptation procedures.

Second, regarding item transparency, the revised manuscript provides the complete wording of all 22 measurement items in Table 2. These include four items for AI adoption, five for job substitution risk, three for job insecurity, four for organizational AI readiness, and six for career exploration behavior. Presenting the items in the main manuscript allows readers to directly assess the operationalization and content of each construct.

Third, regarding construct alignment, we agree that teachers' individual AI adoption should not be conceptualized as an externally imposed stressor. As already clarified in Section 2.1 (Theoretical Framework), AI adoption is operationalized as teachers' individual use of AI tools, whereas the broader development and diffusion of AI technologies represent changes in their occupational environment. Within the Conservation of Resources (COR) framework, teachers' engagement with AI may increase their awareness of the potential automation of artistic and instructional tasks, making perceived threats to valued professional resources more salient. Job substitution risk and job insecurity capture these perceived occupational concerns, rather than AI adoption itself being treated as an external threat.

To reinforce this conceptual distinction, we have made minor wording adjustments in Sections 2.2 and 3.2 to distinguish AI-related technological change from teachers' self-reported use of AI tools. These clarifications preserve the original theoretical framework while improving consistency between the conceptual arguments and the measurement approach.

We sincerely appreciate your valuable suggestions, which have helped us improve the transparency of our measurement procedures and strengthen the conceptual precision of the manuscript.

**Comment 4: Deepening the Theoretical Framework and Context (Reviewer 1)**
As Reviewer 1 points out, the manuscript would benefit from integrating Career Construction Theory (CCT) to provide a more nuanced view of Career Exploration Behavior (CEB), distinguishing between "threat-driven" and "growth-driven" exploration. Furthermore, please strengthen the contextualization of your study. Explicitly discuss how generative AI uniquely challenges the aesthetic authority and creative identity of *art* teachers compared to educators in other disciplines.

**Response 4:** Dear editor, thank you for your insightful and constructive suggestions. We fully agree that integrating Career Construction Theory (CCT) and highlighting the distinctive professional context of art education can provide a more nuanced understanding of teachers' career exploration behavior.

First, regarding the integration of CCT, the revised manuscript incorporates CCT as a complementary perspective to Conservation of Resources (COR) theory. In Section 2.1 (Theoretical Framework), we distinguish between threat-driven career exploration, which may reflect efforts to protect occupational security and professional identity, and growth-driven exploration, which may reflect curiosity, professional development, and the pursuit of emerging career opportunities. This distinction is further developed in Section 2.2, the Discussion, and Section 5.1 (Theoretical Contribution). Importantly, we clarify that these motivations may coexist and that the present study measures overall career exploration behavior rather than separately identifying its underlying motivations.

Second, regarding the contextualization of art education, the manuscript discusses how generative AI presents particular challenges to art teachers' creative expertise, aesthetic judgment, and professional identity. As elaborated in Sections 2.3, 5 (Discussion), and 5.1, generative AI can produce visual artwork, imitate artistic styles, and support creative and evaluative processes. Unlike technologies primarily associated with routine task automation, these capabilities overlap with activities central to artistic practice and teaching, potentially raising questions about the distinctiveness of art teachers' professional contributions and aesthetic authority.

At the same time, we recognize that these challenges are conceptually relevant to art education but have not been empirically established as more pronounced than those experienced by teachers in other disciplines. Section 5.3 (Limitations and Future Directions) explicitly acknowledges the absence of a cross-disciplinary comparison group and recommends further research to examine these contextual differences.

We sincerely appreciate your valuable guidance. The integration of CCT and the strengthened discussion of art-specific professional concerns have helped us develop a more balanced theoretical interpretation and clarify the contextual contribution of our study.

**Comment 5: Control Variables and Sampling (Criterion 3)**

- **Controls:** Reviewer 1 correctly notes that critical control variables, such as professional rank/title and pre-existing digital literacy, are missing. If you have this data, please include it in your models. If you do not, you must explicitly acknowledge the omission of these potential confounding variables as a limitation.
- **Sampling:** Please provide more detail on your snowball/convenience sampling method via social networks and explicitly discuss the potential selection bias this introduces in your limitations section.

**Response 5:** Dear editor, thank you for your careful assessment and constructive suggestions regarding control variables and sampling procedures. We agree that accounting for relevant individual characteristics and transparently describing the recruitment process are important for evaluating the robustness and generalizability of our findings.

First, regarding control variables, we would like to clarify that the study has already incorporated several demographic and professional characteristics into the analysis. Specifically, as reported in Section 4.4 (Moderating Effect), gender, age, educational attainment, teaching experience, school level, and weekly frequency of AI use were included as control variables. The positive interaction between AI adoption and organizational AI readiness remained statistically significant after adjusting for these characteristics.

Nevertheless, we recognize that professional rank/title and pre-existing digital literacy/AI proficiency were not included as control variables. These characteristics may be associated with teachers' perceived job security, technology adoption, and career exploration, and their omission may introduce unmeasured confounding. This limitation is explicitly acknowledged in the fourth paragraph of Section 5.3 (Limitations and Future Directions), where we also recommend that future studies directly measure these characteristics, incorporate them as theoretically relevant covariates, and examine the robustness of the findings after adjustment.

Second, regarding sampling procedures, Section 3.1 (Data Collection) describes the recruitment process in detail. The study involved 30 universities across eastern, central, and western China. Participants were recruited through a non-probability approach combining convenience sampling and referrals through existing social networks. Specifically, we contacted individuals working at the selected institutions, explained the study procedures, and invited them to share the questionnaire with art teachers at their universities. Of the 500 questionnaires distributed, 439 responses were received, and 381 valid responses were retained after screening.

We also recognize that recruitment through personal networks and referrals may introduce selection bias, as teachers with stronger social connections or greater engagement with technology may have been more likely to participate. This concern is explicitly discussed in the second paragraph of Section 5.3, together with the implications for sample representativeness and the generalizability of the findings. We accordingly recommend more diverse recruitment strategies in future research.

We sincerely appreciate your valuable guidance. The current manuscript thus distinguishes between the demographic and professional characteristics already controlled for in the moderation analysis and the additional potential confounders that remain unaddressed. It also provides a transparent account of the sampling procedures and their limitations, allowing readers to evaluate the findings within the appropriate methodological context.

**Comment 6: Clarification of Policy Documents (Reviewer 2)**
In the practical implications, you mention the development of comprehensive 'AI in Education Transformation Support Guidelines.' Please clarify if this refers to an existing Chinese policy document (and cite it accordingly) or if this is a hypothetical recommendation you are proposing.

**Response 6:** Dear editor, thank you for your careful reading and helpful suggestion. We agree that the expression “AI in Education Transformation Support Guidelines” could be interpreted as the official title of an existing Chinese policy document.

Our original intention was to propose a direction for future policy development rather than to refer to an existing policy or officially issued guidelines.

To eliminate this ambiguity, we have revised Section 5.2 (Practical Implications) by removing the potentially misleading title and replacing it with a clearer recommendation for developing practical guidance to support teachers during the integration of AI into art education. The revised paragraph specifies possible institutional support measures, including psychological support, AI ethics training, and professional development opportunities. It also recommends considering the integration of AI literacy and application skills into art teacher preparation, professional qualification requirements, and relevant curriculum standards.

As this is a proposed policy recommendation rather than an existing Chinese policy document, no citation to a specific policy document is applicable. The revised wording now makes this distinction explicit.

We sincerely appreciate your valuable comment, which has helped us improve the clarity and precision of our practical recommendations.

**Reviewer #1:**

**Comment 1:** Deepening the Theoretical Framework: The study would benefit significantly from integrating Career Construction Theory (CCT). While the COR theory provides a solid foundation for explaining the stress and coping mechanism, CCT (Savickas, 2013) offers a more nuanced lens for understanding career exploration behavior (CEB)itself. Currently, CEB is treated as a monolithic, adaptive outcome. CCT would help you theoretically distinguish between "threat-driven CEB" (a reactive, anxiety-based search for alternatives) and "growth-driven CEB" (a proactive, curiosity-based exploration aligned with self-concept). Incorporating this distinction in the Introduction (when introducing CEB) and in the Discussion (when interpreting results) would greatly enhance the theoretical depth and precision of your arguments. Please cite relevant literature, such as Savickas (2013) and other key papers on CCT and proactive career behaviors.

**Response 1:** Dear reviewer, thank you for your insightful and constructive suggestion. We agree with your observation that our original manuscript relied predominantly on Conservation of Resources (COR) theory to explain career exploration behavior (CEB) as a response to AI-related occupational threats, without sufficiently considering the diverse motivations underlying such behavior. Your recommendation to integrate Career Construction Theory (CCT) provides a valuable opportunity to deepen our theoretical framework and refine the interpretation of CEB.

In response to your suggestion, we have revised several sections of the manuscript to incorporate CCT as a complementary theoretical perspective while retaining COR theory as the primary framework for explaining resource-related psychological mechanisms. Specifically, we have made the following revisions.

First, we have refined the Introduction and substantially expanded Section 2.1 (Theoretical Framework). In the Introduction, we now acknowledge that career exploration may reflect both threat-driven efforts to manage occupational uncertainty and growth-driven efforts to pursue professional development opportunities. To maintain conceptual clarity and avoid redundancy, we have concentrated the systematic discussion of COR theory and CCT in Section 2.1. Drawing on relevant literature, including Savickas (2013), Savickas and Porfeli (2012), and Rudolph et al. (2017), we clarify the complementary roles of these two theories. While COR theory explains resource-related threat perceptions and coping responses, CCT provides a broader perspective on career adaptation and vocational self-construction. This integration allows us to distinguish conceptually between threat-driven and growth-driven career exploration.

Second, we have incorporated CCT perspectives into Sections 2.2–2.4 to strengthen the theoretical reasoning underlying our hypotheses. Specifically, Section 2.2 now recognizes that the association between AI adoption and CEB may involve both threat-related and growth-oriented motivations. Section 2.3 discusses how perceived job substitution risk may be associated with threat-driven career exploration as art teachers reconsider their vocational identities and future career directions. Section 2.4 further interprets career exploration as an adapting response to occupational uncertainty, while acknowledging that such behavior is not necessarily motivated exclusively by job insecurity. These additions strengthen the theoretical coherence of our hypotheses without altering the original research model.

Third, we have refined the Discussion and Theoretical Contribution sections to provide a more nuanced interpretation of our findings. The positive indirect associations involving job substitution risk and job insecurity are now discussed as being consistent with the possibility of threat-driven career exploration. At the same time, we acknowledge that career exploration may also reflect growth-oriented motivations, particularly when art teachers investigate emerging opportunities in AI-assisted artistic creation, digital education, and interdisciplinary professional development. This distinction is especially relevant to art education, where generative AI may simultaneously challenge established professional expertise and create new opportunities for career development. Accordingly, the revised discussion recognizes that similar career exploration behaviors may have different underlying motivations rather than treating CEB as a homogeneous coping response.

Finally, we have clarified the measurement limitations of this theoretical distinction in Section 5.3 (Limitations and Future Directions). Although CCT provides a conceptual basis for distinguishing threat-driven and growth-driven exploration, our existing measure assesses overall career exploration behavior rather than these specific motivations. Therefore, we do not claim to have empirically identified or tested two distinct types of CEB. Instead, we present this distinction as a theoretical interpretation and recommend that future research employ motivation-specific measures and longitudinal designs to examine the potentially different antecedents and outcomes of these exploratory orientations.

These revisions have strengthened the theoretical depth, conceptual precision, and coherence of our manuscript. By integrating COR theory with CCT, we now offer a more differentiated understanding of Chinese art teachers' career exploration in the context of AI-related technological change.

We sincerely appreciate your valuable suggestion, which has helped us strengthen both the theoretical framework and the interpretation of our findings.

**Comment 2:** Clarifying and Amplifying the Novel Contribution: The core mediated pathway of "AI adoption → job insecurity → outcome" is not novel in the broader management and applied psychology literature. The incremental value of your study lies primarily in (a) focusing on career exploration behavior as a nuanced outcome, and (b) introducing organizational AI readiness as a critical boundary condition. To maximize the paper's impact, you must more forcefully articulate and contextualize this contribution within the specific domain of art education. Currently, the "art teacher" context is somewhat relegated to background. Please deepen the analysis: How does AI uniquely challenge the aesthetic authority, creative identity, and evaluative role of an art teacher compared to teachers of other subjects? Discuss how generative AI tools for art creation directly threaten the core of their professional expertise. Explicitly linking the mediators (job substitution risk, insecurity) to these art-education-specificthreats will make your model and findings much more distinctive and compelling.

**Response 2:** Dear reviewer, thank you for your insightful and constructive comments. We agree with your observation that the general relationship between AI adoption and job insecurity is not inherently novel in the broader management and applied psychology literature. We also appreciate your emphasis on career exploration behavior (CEB), organizational AI readiness, and the distinctive professional context of art education as the key contributions of our study.

In response to your suggestion, we have made targeted revisions to clarify the study's distinctive value and strengthen its connection to the specific professional challenges faced by art teachers.

First, we have refined the Introduction and Sections 2.2–2.4 to better highlight the distinctive context of art education. We now explain more explicitly how generative AI, through its capabilities in image generation and artistic style imitation, may challenge art teachers' creative expertise, aesthetic judgment, and evaluative roles. These challenges may raise concerns about the continuing value of their professional skills and responsibilities, providing a more context-specific rationale for the proposed relationships involving job substitution risk and job insecurity. We have also clarified the relevance of CEB as a career-related behavioral response that extends beyond commonly examined outcomes such as anxiety and turnover intentions.

Second, we have strengthened Section 2.6 to clarify the relevance of organizational AI readiness to art education. In particular, we now emphasize that institutional AI readiness may involve not only technological infrastructure and professional training but also support for AI-assisted artistic creation and guidance on evaluating AI-generated artworks. Such resources may be particularly relevant to art teachers' professional adaptation because questions of artistic originality, creative expertise, and aesthetic evaluation are closely connected to their occupational roles. We have retained the original COR-based threat-buffering expectation while discussing the observed positive moderating effect in the Discussion, where CCT offers a complementary interpretation involving possible growth-oriented career exploration.

Third, we have refined the Discussion and Section 5.1 (Theoretical Contribution) to articulate the study's incremental contributions more clearly. Rather than emphasizing the general AI adoption–job insecurity relationship as a novel mechanism, we now place greater emphasis on CEB as a meaningful career-development outcome, the art-education-specific context of perceived occupational threats, and organizational AI readiness as an important boundary condition. To avoid redundancy, we have kept the Introduction focused on the research background and gap, while concentrating the discussion of theoretical contributions in Section 5.1.

We have also acknowledged that aesthetic authority, creative identity, and evaluative expertise were not directly measured and that our data do not establish differences between art teachers and teachers of other subjects. Accordingly, these professional characteristics are presented as contextual interpretations rather than additional empirically verified mechanisms.

These targeted revisions have clarified the distinctive contribution of our study while preserving its original theoretical framework and empirical focus. Thank you again for your valuable suggestion, which has helped us improve the manuscript's theoretical positioning and relevance to art education.

**Comment 3:** Addressing Omitted Control Variables: The list of control variables (age, work experience) is insufficient. Several other demographic and professional factors are likely to confound the relationships you are testing. Most notably, you have not controlled for professional rank/title (e.g., lecturer, associate professor) and digital literacy/AI proficiency. A teacher's rank influences their job security perceptions and career mobility options. Their pre-existing digital literacy would directly affect their perception of AI as a threat versus a tool, thereby influencing both the mediators and the outcome. Please justify the choice of current control variables and acknowledge the omission of these potential confounds as a limitation in the discussion. If possible, conducting additional analyses including these variables (if data is available) would significantly strengthen the robustness of your conclusions.

**Response 3:** Dear reviewer, thank you for your thoughtful and constructive suggestion. We appreciate your comments regarding the selection of control variables and the potential relevance of professional rank/title and pre-existing digital literacy/AI proficiency to the relationships examined in our study.

The selection of control variables in our study was informed by previous AI-related research, including Brougham and Haar (2018), Fan et al. (2025), and Presbitero and Teng-Calleja (2023). As reported in Section 4.4, the moderation analysis included six demographic and professional characteristics as control variables: gender, age, educational attainment, teaching experience, school level, and weekly frequency of AI use.

In response to your suggestion, we have made two targeted revisions to the manuscript.

First, we have refined Section 4.4 (Moderating Effect) to clarify the control variables included in the analysis and their corresponding results. Specifically, gender, age, educational attainment, and teaching experience were not statistically significant, and none of the individual coefficients for school level or weekly frequency of AI use reached statistical significance. The positive interaction between AI adoption and organizational AI readiness remained statistically significant after adjustment for these demographic and professional characteristics.

Second, we have expanded Section 5.3 (Limitations and Future Directions) to acknowledge the omission of professional rank/title and pre-existing digital literacy/AI proficiency. As you noted, professional rank may be associated with teachers' perceived job security and career mobility, while digital literacy and AI proficiency may influence their perceptions of AI-related occupational threats and career exploration behavior. We have therefore identified these characteristics as relevant considerations for future research and recommended their inclusion in subsequent analyses to further assess the robustness of the proposed relationships.

We recognize the importance of your recommendation regarding additional covariate adjustments. Although the current analysis included several demographic and professional characteristics, professional rank/title and digital literacy/AI proficiency were not included as additional controls. We have acknowledged this limitation in the revised manuscript and identified further examination of these factors as a direction for future research.

Thank you again for your valuable suggestion.

**Reviewer #2**

**Comment 1:** My biggest concern is that the authors do not sufficiently operationalize the concepts used in the study. In the methods section, the authors provide one or two examples of survey items used for each construct. To help readers assess the validity of their findings, I would recommend that the authors describe their survey items in depth. Right now, the survey questionnaire is available as an appendix. I recommend moving this to the main text.

**Response 1:** Dear reviewer, thanks for your valuable and constructive suggestion. We agree with your observation that including only representative survey items in the Methods section may not enable readers to adequately evaluate the operationalization and measurement properties of the study constructs.

As you noted, the complete set of measurement items was provided separately in the questionnaire appendix. However, we recognize that presenting the full item wording in the main manuscript would make the measurement procedures more transparent and facilitate readers' assessment of construct validity.

In response to your recommendation, we have revised Section 3.2 (Measures) and incorporated all 22 measurement items into a new table in the main text (Table 2). Specifically, the table presents four items measuring AI adoption, five items measuring job substitution risk, three items measuring job insecurity, four items measuring organizational AI readiness, and six items measuring career exploration behavior. Each item is presented with its corresponding measurement code, allowing readers to relate the questionnaire content to the confirmatory factor analysis and reliability results.

We have also refined the accompanying descriptions in Section 3.2 to clarify the operationalization of each construct and retained the original references for the measurement scales. In addition, the section specifies the seven-point Likert response format and the three-month reference period for career exploration behavior.

These revisions allow readers to examine the distinction between perceived job substitution risk and job insecurity, the organizational resources represented by AI readiness, and the specific career exploration activities assessed in this study. Importantly, the career exploration items measure observable exploratory behavior rather than separately assessing threat-driven or growth-driven motivations, consistent with the scope of our empirical model.

The revision concerns the presentation and transparency of the existing measurement instruments; the original survey items and empirical model remain unchanged.

We sincerely appreciate your recommendation, which has helped us improve the clarity, transparency, and accessibility of the measurement information in the manuscript.

**Comment 2:** Similarly, all survey items rely on teacher perceptions, raising questions about to what extent their answers reflect their actual actions, behaviors, and workplace environments. A case in point is organizational AI readiness. The survey item used is “our institution has sufficient financial resources to support AI-assisted art teaching and artistic projects”. What constitutes sufficient resources? The authors should address this limitation and discuss potential bias.

**Response 2:** Dear reviewer, thank you for your thoughtful and constructive comment. We appreciate your concern regarding the reliance on teacher-reported measures and the extent to which these measures reflect actual behaviors and organizational conditions. Your observation concerning the interpretation of “sufficient financial resources” in the organizational AI readiness scale is particularly valuable.

First, we have added a new subsection, Section 3.4 (Common Method Bias Test), to report an assessment of potential common method bias. Specifically, Harman's single-factor test was conducted using all 22 measurement items from the 381 respondents. The largest eigenvalue of the item correlation matrix was 6.173, accounting for 28.06% of the total variance, below the commonly used 50% threshold. In addition, the single-factor model exhibited poor fit (χ²(209) = 2564.49, CFI = .404, TLI = .341, RMSEA = .172, SRMR = .150). These findings suggest that no single factor dominated the survey responses, although the test cannot completely rule out potential common method bias.

Second, we have clarified the operationalization of organizational AI readiness in the Measures section. As our study focuses on the relationships between teachers' individual perceptions and career exploration behavior, organizational AI readiness was measured in terms of teachers' perceived institutional preparedness to support AI-assisted art teaching. The four items capture teachers' perceptions of institutional financial resources, AI-related expertise, technological infrastructure, and leadership support. Accordingly, “sufficient financial resources” refers to respondents' perceived adequacy of institutional financial support for AI-assisted art teaching and artistic projects, rather than an assessment based on standardized institutional budgetary criteria.

Third, we acknowledge the measurement limitations associated with teacher-reported data. Although teachers' perceptions are central to the research questions, these perceptions may not fully correspond to actual institutional conditions. Similarly, self-reported career exploration may differ from independently documented behavior. We therefore recognize the potential influence of perceptual and response-related biases and identify the use of multiple data sources, including institutional records and independently assessed behavioral indicators, as an important direction for future research.

We believe these revisions clarify the individual-level perceptual focus of the study, provide additional evidence regarding potential common method bias, and offer a more transparent interpretation of the measurement approach and its limitations.

Thank you again for your valuable suggestion, which has helped us strengthen the methodological clarity and rigor of the manuscript.

**Comment 3:** The authors theorize that AI adoption leads teachers to explore alternative career options. Could the relationship be the other way around? Could teachers who are already looking for alternative careers be more proactive or tech-savvy, making them more likely to adopt AI technology in the first place? This is just a possibility but the causal direction of these variables needs to be further examined to justify their theoretical framework.

**Response 3:** Dear reviewer, thank you for your thoughtful and constructive suggestion. We agree with your observation that the relationship between AI adoption and career exploration behavior may be bidirectional. In particular, teachers who are already exploring alternative career opportunities may be more proactive in their professional development or possess stronger digital competencies, making them more inclined to adopt AI technologies.

The proposed direction of the relationships in our study is grounded primarily in Conservation of Resources (COR) theory. From this perspective, teachers' engagement with AI may heighten their awareness of potential threats to valued professional resources, including job substitution risk and employment insecurity, which may be associated with career exploration behavior. This provides the theoretical rationale for the proposed model.

As already acknowledged in Section 2.1 (Theoretical Framework), the ordering of the variables is theoretically specified, while the cross-sectional data cannot establish their temporal or causal direction. We recognize that the alternative explanation you raised is also plausible and warrants further investigation.

In response to your suggestion, we have further refined the first paragraph of Section 5.3 (Limitations and Future Directions) to address this possibility more explicitly. Specifically, we now discuss how teachers who are already engaged in career exploration, particularly those with proactive career development orientations or stronger pre-existing digital competencies, may be more likely to adopt AI tools. We have also highlighted the value of longitudinal research involving repeated measurements of AI adoption and career exploration to examine their potential bidirectional relationship.

The current findings therefore provide evidence of associations consistent with the proposed theoretical framework, rather than establishing a definitive causal sequence.

Beyond improving the present manuscript, your comment has provided valuable inspiration for our future research. We are already planning a follow-up study that builds on this alternative perspective, with particular attention to the potential reciprocal relationship between career exploration and AI adoption. We believe that examining this possibility could further deepen our understanding of teachers' career adaptation in the context of technological change. We would greatly value your continued insights and guidance as this line of research develops.

Thank you again for your insightful and encouraging feedback. Your suggestion has not only helped us refine the interpretation of our current findings but has also opened up a meaningful direction for our subsequent research.

**Comment 4:** In the conclusion section, the authors discuss “the development of comprehensive 'AI in Education Transformation Support Guidelines,'” as a practical implication for policymakers. Please define what ‘AI in Education Transformation Support Guidelines’ are. If this is referring to an existing policy document or a resource, please cite accordingly.

**Response 4:** Dear reviewer, thank you for your careful reading and constructive suggestion. We agree with your observation that the expression “AI in Education Transformation Support Guidelines” could be interpreted as the official title of an existing policy document or resource.

Our original intention was to propose a possible direction for policy development rather than to refer to an existing policy document. Specifically, we intended to suggest that education policymakers could develop practical guidance to help institutions support teachers during the integration of AI into art education.

In response to your comment, we have revised Section 5.2 (Practical Implications), where this expression appeared. We have removed the potentially misleading title and replaced it with a more explicit description of the proposed policy guidance. The revised paragraph now specifies that such guidance could recommend institutional support measures, including psychological support, AI ethics training, and professional development opportunities. We have also clarified our recommendation concerning the incorporation of AI literacy and application skills into art teacher preparation, professional certification, and relevant curriculum standards.

As the recommendation concerns a proposed policy approach rather than an existing policy document, no citation to a specific policy resource is intended. The revised wording makes this distinction clear while retaining the practical implications of our findings.

We sincerely appreciate your helpful comment, which has enabled us to improve the precision and clarity of our policy recommendations.
