# Supplementary material for: Artificial intelligence (AI) adoption and Chinese art teachers’ career exploration behavior: The roles of job substitution risks, job insecurity, and organizational AI readiness
Source: PLoS One. 2026 Nov 6;21(11):e0362478. doi: 10.1371/journal.pone.0362478 (PMC13742945; doi:10.1371/journal.pone.0362478)
Supplement: Supplementary file 2 [file pone.0362478.s003.docx]

Dear Editor,

Thank you for your careful evaluation of our revised manuscript and for providing constructive comments. We have carefully addressed your three comments and made corresponding revisions to improve the mathematical consistency, clarity of model specification, and transparency of statistical reporting. All revisions have been ***highlighted in yellow*** in the revised manuscript for ease of reference.

Below, we provide point-by-point responses to your comments and explain the corresponding revisions.

**Editor’s Comments**

**Comment1:** Mathematical Consistency in the Moderation Analysis (Criterion 3)

In Section 4.4 and Figure 2, you report the simple slopes for the relationship between AI adoption and career exploration at different levels of organizational AI readiness (OAR). The reported slopes are 0.002 (Low OAR, -1 SD), 0.272 (Mean OAR), and 0.542 (High OAR, +1 SD). This indicates a perfectly symmetric change in the slope of exactly 0.270 per 1 SD of OAR.

However, the interaction coefficient is reported as B = 0.36 in Table 7, and B = 0.34 in the text for the separate simple slope model. If the interaction coefficient is 0.34 or 0.36, and the standard deviation of OAR is 0.89 (as reported in Table 4), the change in slope per 1 SD should be approximately 0.30 to 0.32, not 0.27. Please review your Mplus output and calculations for accuracy to ensure that the interaction coefficients and simple slopes are mathematically consistent. If the variables were standardized or transformed prior to this specific analysis, please state this explicitly in the Methods or Results section.

**Response 1:** Dear Editor, thank you for your careful observation. We agree that the interaction coefficients and simple slopes reported in the previous version were not mathematically consistent. To address this issue, we carefully re-examined the Mplus output and re-estimated the mediation and moderation effects simultaneously within a single integrated latent-variable structural equation model using robust maximum likelihood estimation (MLR), numerical integration, and the XWITH procedure. All interaction estimates and simple slopes reported in the revised manuscript are now derived from this same model.

The revised model yielded a significant positive interaction between AI adoption and organizational AI readiness (B = 0.362, SE = 0.091, p < .001). The latent standard deviation of organizational AI readiness was 0.808, whereas the standard deviation of 0.89 reported in Table 4 refers to its observed composite score. Using the latent standard deviation, the unstandardized conditional slopes at low (−1 SD), mean, and high (+1 SD) levels of organizational AI readiness were −0.020, 0.272, and 0.565, respectively. These estimates were calculated directly from the interaction coefficient and the corresponding latent moderator values.

For graphical presentation, the conditional slopes were transformed to represent changes in the mean career exploration behavior item score associated with a one-standard-deviation increase in latent AI adoption. Specifically, each latent-scale slope was multiplied by the latent standard deviation of AI adoption (1.053) and the mean factor loading of career exploration behavior (0.922). The resulting slopes were −0.019, 0.264, and 0.548 at low, mean, and high organizational AI readiness, respectively.

The difference between adjacent slopes on the transformed scale was approximately 0.284, consistent with the calculation 0.362 × 0.808 × 1.053 × 0.922 ≈ 0.284. Thus, the revised interaction coefficient and conditional slopes are mathematically consistent when evaluated on their respective scales using the unrounded estimates.

Accordingly, we have revised Section 3.3 to clarify the estimation and scale-transformation procedures, updated the moderation results in Section 4.4 and Table 7, and revised Figure 2 to reflect the conditional associations derived from the integrated model. The previous estimates obtained from the separately specified model have been replaced with results from the integrated model.

We appreciate your comment, which has helped improve the mathematical consistency and transparency of our statistical reporting.

**Comment 2:** Clarity of Model Specification (Criterion 3)

The text mentions that a "separately specified latent-variable model" was used for the simple slope analysis, and Table 7 reports a moderation model that includes the mediators (JSR and JI) as predictors. It is currently unclear whether the mediation (Table 6) and moderation (Table 7) were tested as a single, integrated moderated-mediation model in Mplus, or as separate piecemeal regressions. To meet PLOS ONE’s rigorous technical standards, it is highly recommended to estimate the full moderated-mediation model simultaneously to avoid biased estimates. Please clarify your analytical approach in the Methods section and ensure that the coefficients reported across the text, tables, and figures are derived from a consistent model specification.

**Response 2:** Dear Editor, thank you for your important comment. We agree that the analytical approach was insufficiently clear in the previous version, particularly regarding whether the mediation and moderation effects were estimated in the same model. To address your concern, we re-estimated the hypothesized relationships simultaneously using a single integrated latent-variable structural equation model in Mplus 7.4, rather than relying on separately specified models.

Specifically, the revised model simultaneously estimated the associations of AI adoption with job substitution risk and job insecurity, the association between the two mediators, and their respective associations with career exploration behavior. Organizational AI readiness was included as a predictor of the mediators and career exploration behavior, while its latent interaction with AI adoption was specified to moderate only the direct association between AI adoption and career exploration behavior. The model was estimated using robust maximum likelihood estimation (MLR), numerical integration, and the XWITH procedure for the latent interaction. All specified control variables were included in the structural equations for the endogenous constructs.

The three specific indirect associations, their total, and the conditional direct and total associations were calculated from the estimated coefficients of this integrated model. The simple slope analyses were also based on the same model rather than a separately estimated model. Accordingly, Section 4.3 reports the structural path estimates, while Table 6 summarizes the total, direct, and indirect effects. Table 7 presents the moderation estimates, and Figure 2 illustrates the conditional associations between AI adoption and career exploration behavior at different levels of organizational AI readiness. These results were all derived from the same fitted model rather than separate regression analyses.

We have revised Section 3.3 (Data Analysis Strategy) to clarify the integrated model specification and estimation procedures. We have also updated Sections 4.3 and 4.4, Tables 6 and 7, and Figure 2 to ensure consistency in the reported estimates and their interpretation. The previous reference to a "separately specified latent-variable model" has been removed.

We appreciate your recommendation, which has improved the clarity, consistency, and transparency of our analytical approach.

**Comment3:** Reporting of Control Variables (Criterion 3)

While the text in Section 4.4 states that control variables (gender, age, educational attainment, teaching experience, school level, and weekly frequency of AI use) were included in the moderation analysis, these variables and their coefficients are omitted from Table 7. For transparency and completeness, please either include the control variables and their estimates directly in Table 7 or add a clear table note indicating that the reported estimates are adjusted for these specific controls.

**Response 3:** Dear Editor, thank you for your constructive suggestion regarding the reporting of control variables. We agree that explicitly identifying the control variables is important for ensuring transparency and completeness in the presentation of the moderation results.

Following the second reporting option you suggested, we have revised the note to Table 7 to explicitly identify the control variables included in the model, without presenting their individual coefficient estimates in the table. Specifically, all reported estimates were adjusted for gender, age, educational attainment, teaching experience, institutional level, and weekly frequency of AI use. These control variables were consistently included in the structural equations for all three endogenous constructs within the integrated latent-variable model.

To further clarify the model specification, the revised table note states that categorical covariates were dummy-coded, with male, bachelor's degree or below, junior/vocational college, and AI use fewer than three times per week serving as the respective reference categories. Age and teaching experience were grand-mean centered. The inclusion of these controls is also explicitly described in Section 3.3 (Data Analysis Strategy).

Accordingly, Table 7 now clearly indicates that all reported moderation estimates were adjusted for the specified demographic and professional characteristics, while maintaining a concise presentation of the principal model coefficients.

We appreciate your suggestion, which has helped improve the transparency and clarity of our statistical reporting.
