# Supplementary material for: Artificial intelligence (AI) adoption and Chinese art teachers’ career exploration behavior: The roles of job substitution risks, job insecurity, and organizational AI readiness
Source: PLoS One. 2026 Nov 6;21(11):e0362478. doi: 10.1371/journal.pone.0362478 (PMC13742945; doi:10.1371/journal.pone.0362478)
Supplement: Supplementary file 3 [file pone.0362478.s004.docx]

Dear Editor,

Thank you for your careful evaluation of our revised manuscript and for providing constructive comments. We have carefully addressed both comments and made corresponding revisions to improve the mathematical consistency and transparency of our statistical reporting. All revisions have been ***highlighted in yellow*** in the revised manuscript for ease of reference.

Below, we provide point-by-point responses to your comments and explain the corresponding revisions.

**Editor’s Comments**

**Comment1:** Harmonize the Main Effect and the Simple Slope at the Mean

In a mean-centered moderation model, the main effect of the predictor is mathematically identical to the simple slope at the mean of the moderator.

On page 25 (and in Table 7), you report the main effect of AI adoption as B = 0.27.

On page 26, you report the simple slope at the mean as B = 0.26.

This discrepancy occurs because you reported the latent estimate for the main effect, but the transformed estimate for the simple slope. To prevent reader confusion, please report the estimates consistently. We highly recommend reporting the unstandardized latent estimates (-0.020, 0.272, 0.565) in the text for the simple slope analysis so that they perfectly match the coefficients in Table 7. You can then reserve the transformed values strictly for the visual representation in Figure 2 (and explicitly state in the Figure 2 caption that the Y-axis represents transformed item-score scales).

**Response 1:** Dear Editor, thank you for your careful observation. We agree that the previous version used different reporting scales for the main effect of AI adoption and the simple slope at the mean of organizational AI readiness, which could cause confusion.

In response, we have revised Section 4.4 to consistently report the unstandardized latent-variable estimates for the simple slopes at low, mean, and high levels of organizational AI readiness: B = −0.02 (SE = 0.11, p = .853), B = 0.27 (SE = 0.08, p < .001), and B = 0.57 (SE = 0.10, p < .001), respectively. The simple slope at the moderator mean (B = 0.27) is now consistent with the main-effect coefficient reported in Table 7 (B = 0.27).

We have also clarified the reporting scale in the note to Table 7 and revised the caption of Fig. 2 to specify that the Y-axis represents career exploration behavior on the transformed mean-item-score scale. The transformed values are retained for graphical presentation.

These revisions improve the consistency and clarity of the statistical reporting. We appreciate your constructive guidance.

**Comment 2:** Include the Transformation Details in the Manuscript

If a reader attempts to verify your moderation math using the manuscript alone, they will multiply the interaction coefficient (B = 0.36, Table 7) by the standard deviation of OAR (SD = 0.89, Table 4), expecting a slope change of ~0.32 per SD. Because the manuscript does not mention that the latent SD of OAR is 0.808, readers will assume your math is incorrect.

Please take the excellent explanation you provided in "Response 1" of your rebuttal letter and integrate a brief version of it into the manuscript (e.g., in Section 3.3, Section 4.4, or as a detailed note under Table 7). Specifically, please explicitly state:

That the latent standard deviation of OAR (0.808) was used for the simple slope calculations, rather than the observed composite score SD (0.89).

The exact transformation formula/multipliers used for the graphical presentation (multiplying by the latent SD of AI adoption [1.053] and the mean factor loading of career exploration behavior [0.922]).

**Response 2:** Dear Editor, thank you for your important comment. We agree that explicitly reporting the latent standard deviation used in the simple slope calculations and the transformation procedure for graphical presentation is essential for ensuring the transparency and reproducibility of our statistical analyses.

In response, we have incorporated these details into the simple slope analysis paragraph in Section 4.4 (Moderating Effect). We chose this location because it directly presents the conditional effects of AI adoption at different levels of organizational AI readiness (OAR). Placing the calculation details alongside the corresponding results allows readers to understand and verify the simple slope estimates and their graphical representation without interrupting the methodological overview in Section 3.3 or adding excessive detail to the note accompanying Table 7.

Specifically, we now clarify that the low, mean, and high levels of OAR were defined using its latent standard deviation (SD = 0.808), rather than the observed composite-score standard deviation reported in Table 4 (SD = 0.89). We have also specified that, for graphical presentation in Fig. 2, the unstandardized latent-variable simple slopes were transformed to the mean-item-score scale using the formula B_plot = B_latent × SD_AIA × λ̄_CEB, where SD_AIA is the latent standard deviation of AI adoption (1.053) and λ̄_CEB is the mean factor loading of career exploration behavior (0.922).

These revisions clarify the distinction between the latent-variable estimates reported in the text and the transformed slopes used for graphical presentation, while providing the information needed to independently verify the calculations.

We sincerely appreciate your constructive guidance, which has helped improve the clarity and reproducibility of our statistical reporting.
